# Long-Term Exposure to Phenanthrene Induced Gene Expressions and Enzyme Activities of *Cyprinus carpio* below the Safe Concentration

**DOI:** 10.3390/ijerph19042129

**Published:** 2022-02-14

**Authors:** Xin Kang, Dongpeng Li, Xiaoxiang Zhao, Yanfeng Lv, Xi Chen, Xinshan Song, Xiangyu Liu, Chengrong Chen, Xin Cao

**Affiliations:** 1 Textile Pollution Controlling Engineering Center of Ministry of Environmental Protection, College of Environmental Science and Engineering, Donghua University, Shanghai 201620, China; cathy3449@163.com (X.K.); lddest@163.com (D.L.); zxx@dhu.edu.cn (X.Z.); lvyf_up@outlook.com (Y.L.); newmountain@163.com (X.S.); 2 Agricultural Genomics Institute at Shenzhen, Chinese Academy of Agricultural Sciences, Shenzhen 518124, China; chenxi02@caas.cn; 3 Australian Rivers Institute, School of Environment and Science, Griffith University, Brisbane, QLD 4111, Australia; xiangyu.liu2@griffithuni.edu.au (X.L.); c.chen@griffith.edu.au (C.C.)

**Keywords:** *Cyprinus carpio*, phenanthrene ecotoxicology, cytochrome P4501A, 7-ethoxylesorufin O-deethylase, glutathione S-transferase

## Abstract

Phenanthrene (PHE) is a typical compound biomagnified in the food chain which endangers human health and generally accumulates from marine life. It has been listed as one of the 16 priority PAHs evaluated in toxicology. In order to evaluate the changes of *CYP1A GST* mRNA expression and EROD GST enzyme activity in carp exposed to lower than safe concentrations of PHE. Long-term exposure of carp to PHE at lower than safe concentrations for up to 25 days. The mRNA expression level and cytochrome P450 (CYP1A/EROD (7-Ethoxylesorufin O-deethylase)) and glutathione S-transferase (GST) activity were measured in carp liver and brain tissue. The results showed that PHE stress induced low-concentration induction and high-concentration inhibition of *CYP1A* expression and EROD enzyme activity in the liver and brain of carp. In both two organs, GST enzyme activity was also induced. However, the expression of *GST* mRNA was first induced and then inhibited, after the 15th day. These results indicate that long-term exposure to PHE at lower than safe concentrations still poses a potential threat to carp’s oxidase system and gene expression.

## 1. Introduction

Polycyclic aromatic hydrocarbons (PAHs) are a widespread pollutant which derived from the incomplete combustion of various fossil fuels and hydrocarbons, such as coal and petroleum. Due to the stable chemical properties and harmful effects, PAHs have become persistent organic pollutants (POPs) and threaten environment and even human beings. Especially, PAHs are known to induce stress and affect marine organisms health [1]. According to the combined structure of benzene rings, PAHs can be divided into two categories: fused ring type and non-fused ring type. Phenanthrene (PHE, C_14_H_10_) is a representative of the fused ring PAHs [2]. It is a low-molecular-weight PAH-composed of three fused benzene rings with bay-area and K-area, and its carcinogenicity is closely related to K-area [3]. Due to the small molecular weight of PHE, it is more easily bio-amplified than other PAHs and produces toxic effects [4] like acute lethal effect on aquatic organisms at individual level [5] which further limits individual development [6], reproduction and other behaviors [7,8]. At the levels of cells, tissues, and organs, PHE can cause histopathological changes in organisms [9,10]. Carp **(*Cyprinus carpio*)**, as a traditional freshwater economic fish in China, has been widely used in the ecotoxicity tests of environmental pollutants [11,12]. However, the study of carp genes expressions and enzymes activities after low dose PHE exposure were seldom reported. Therefore, it is necessary to explore the molecular response of carp-specific genes and characteristic metabolic enzymes in PHE pollution stress.

Current studies on low-molecular-weight PAHs in China have mainly focused on their sources and distributions in rivers and lakes [13,14]. Concentrations of PAHs in the waters of Hangzhou, Qiantang River, and Daya Bay reached 989~9663 ng/L, 70.3~1844.4 ng/L and 10984~19445 ng/L, respectively. The effects of high concentrations of PAHs on aquatic organisms were extensively explored [15,16], but the effects of pollutants below safe concentrations on aquatic organisms were still elusive. In the study, the acute toxicity of PHE to carp was tested and the 96-h lethal concentration and safe concentration of PHE to carp were determined. In the experiment, carp were exposed to the PHE at concentrations below safe levels to explore the changes in the activities of characteristic enzymes and characteristic genes expression involved in carp metabolism.

Cytochrome P450 (CYP) enzymes were reported as the biomarkers to estimate the influences of persistent organic pollutants (POPs) on various aquatic organisms [17,18,19]. PAHs, which are known agonists for controlling aromatic hydrocarbon receptors [20,21], could induce the expressions of Phase I and Phase II metabolism enzymes including cytochrome P4501A (CYP1A) and glutathione S-transferase (GST).

Although there have been toxicological studies on PHE stress on aquatic organisms, most of the tested organisms are zebrafish, medaka, tilapia, etc. Moreover, most of the exposure concentrations of phenanthrene are concentrated in high or low concentrations. There are not many articles about carp characteristic gene expression and characteristic enzyme activity changes in the environment of carp long-term exposure to lower than safe concentration of PHE [22,23,24]. Carp is a common freshwater fish in China that can be eaten by humans. It is very important to explore the mechanism of carp being enriched at a safe concentration. This study provides a more complete explanation of the mechanism of action of PHE in carp at lower than safe concentrations.

In this study, the acute toxicity test of PHE was firstly performed on carp, and the PHE concentration group was set for subacute toxicity test according to the measured 96h-LC50, and the safe concentration was calculated to be 1.12 mg/L. Although the toxicity test of PHE to aquatic organisms in natural water has been studied [25], this experiment is higher than that of PHE in natural water but lower than the safe concentration of carp. Long-term exposures were conducted to explore the chronic effects of PHE on carp. The study aimed to explore the activity changes of Phase I and Phase II metabolic characteristic enzymes (cytochrome P450 enzyme (EROD) and glutathione S-transferase (GST) in carp under the exposure to PHE below safe concentration in different organs and determine the changes in the expression levels of characteristic genes of *CYP1A* and *GST*, which controlled the activities of EROD and GST. In addition, the correlations between the expressions of characteristic enzymes (EROD, GST) from metabolic process and the expressions of characteristic genes (*CYP1A*, *GST*) under the exposure to PHE at or below safe concentration were further discussed. We illustrated the responses of carp metabolizing characteristic genes and characteristic enzymes to low concentrations of PHE.

## 2. Materials and Methods

### 2.1. Main Reagents

Phenanthrene (PHE, 95%) was bought from Beijing Braun Technology Ltd. Diethy pyrocarbonate (DEPC), Spin Column Animal Total RNA Purification Kit, and M-MuLV First Strand cDNA Synthesis Kit were obtained from Sangon Biotech (Shanghai, China). Taq Plus PCR MasterMix was bought from Tiangen Biotech (Beijing, China). Agarose was bought from Aladdin. TAE loading buffer and DNA loading buffer were configured according to general methods [26]. Fish 7-ethoxyresorufin-o-deethylase (EROD) kit and fish glutathione S transferase (GST) kit came from Jianglai Biotechnology (Shanghai, China). Fish ELISA kits of the enzymes of EROD and GST were bought from Jianglai Biotechnology (Shanghai, China). Albumin from bovine serum (BSA) was bought from Jiancheng Bioengineering (Nanjing, China).

### 2.2. Fish and Treatment

Carp (*Cyprinus carpio*, body weight (9.0~11.0 g) and body length (8.0 ± 10.0 cm)) were obtained from a fish farm in Songjiang, Shanghai, China. The experimental carp were domesticated in seven groups of 10-L glass tanks under natural conditions for 2 weeks, each group for 10 fish. All tanks were supplied with continuous aeration to maintain nearly saturated dissolved oxygen. Dechlorinated tap water was used at a temperature of 20 ± 2 °C, pH 6.8~7.3, with light intensity of 100 Lux for 10 h/d for 25 d and fed twice a day and clean up metabolites on time to ensure that the mortality rate during domestication is less than 1%, and 4 d before the start of the experiment, ensure that there was no fish death. Three treatment groups were exposed to waterborne PHE in acetone under the concentrations of 0.1, 0.5, and 1.0 mg/L (the final content of acetone was less than 0.5%) In addition, the 0 mg/L PHE as the negative control (dechlorinated water) was established. The parallel groups of each concentration were set in other three 10-L tanks at 20.0 ± 2 °C. After the exposure for 1 days, 5 days, 15 days and 25 days (We define 1–5 days as “early period”), the brain and liver of fish were dissected, placed in 1.5-mL microcentrifuge tubes, frozen in liquid nitrogen immediately, and then stored at −80 °C until further analysis. A part of one sample was ground fully in a vessel with liquid nitrogen to analyze the expressions of relevant genes. Another part of the sample was ground fully with a glass homogenate device with PBS to detect the activities of related enzymes. All samples are homogenized before separation.

### 2.3. Acute Toxicity Test

The PHE dose was set to 5, 10, 20, 30, 40, 50 mg/L (the final content of acetone was less than 0.5%) using the aquatic toxicity test method, and each group was 10 pieces of fish. The pre-test first showed that the carp did not die in 96 h. The highest dose and the lowest dose for all deaths in 24 h. During the test, no feed was fed, semi-static exposure was performed, and the water was changed 50% every day. The activity status of the carp was observed over time and the number of dead fish was counted in time.

The acute toxicity experiment was conducted according to the SECP-Part 12: Fish Acute Toxicity Test (GB/T 31270.12-2014). PHE in eight tanks was set according to equidistant logarithmic concentrations at 5.01, 6.31, 7.94, 10.00, 12.59, 15.85, 19.95, and 25.12 mg/L and there were 10 fish in each tank. Three replicates were arranged for each concentration. In the acute test, after 96 h, the mortality was recorded. The carp were not fed during the test period and water was replaced once a day.

Dead fish were identified according to the following method. Carp were transferred to clear water for 30 s and the fishtail was touched with a glass rod. If there was no visible response, carp were considered to be dead.

The safe concentration (*SC*) is expressed as follows:

(1) $$\left(SC\right)=\frac{\;\left(LC50\left(96h\right)\right)\;}{10}$$

### 2.4. RNA Extraction and Analysis

All samples from the experimental fish were firstly ground into fine powder under liquid nitrogen. Total RNA was extracted with Trizol Reagent (Sangon, China) according to the manufacturer’s instructions. Then the OD (Optical density) values of samples at 260 nm and 280 nm were measured. The RNA sample purity was calculated with Equation (2). Finally, based on the calculation results, the integrity of RNA was detected by 1.5% agarose gel electrophoresis (Figure S1).

The purity of RNA is expressed as:

(2) $$\omega =\frac{A_{260}}{A_{280}}$$

If *ω* is between 1.8 and 2.0, the RNA sample is qualified; if *ω* is less than 1.8, the sample is contaminated; if *ω* is larger than 2.0, the RNA sample is degraded.

### 2.5. Gene Primer Design and Semi-Quantitative RT-PCR (Sq-RT-PCR) Analysis

After RNA extraction, the RNA was reversely transcribed with the cDNA Synthesis Kit (Sangon, China) according to the manufacturer’s protocol (Figures S2 and S3). Then, the mRNA levels were expressed as the ratios relative to the transcription level of the β-action. Primer Premier 5.0 was used to design the primers according to two ends of the target gene based on the sequence of carp *CYP1A* (accession: AB048939.1) and *GST* (accession: LC071505.1) (Table 1).

We performed Sq-RT-PCR to check the changes in *GST* and *CYP1A* mRNA levels in the carp liver and your brain. For Sq-RT-PCR amplification, each reaction included 10 μL Taq Plus PCR MasterMix, 0.4 μL 10 μmol·L^−1^ specific primer F, 0.4 μL 10 μmol·L^−1^ specific primer R and 1 μL cDNA. (all are the configuration in the kit instructions). The reaction conditions are as follows: 94 °C/5 min; 35 cycles of 94 °C/30 s, 55 °C/30 s, 72 °C/30 s; and 72 °C/10 min. We used the β-actin gene as a reference sample for the relative expression levels between normalizations. The product was subject to agarose gel electrophoresis to detect the amplified product, using the UVitec Cambridge scanning system and the software ImageQuant TL (GE Healthcare). All mRNA data represent normalization for any difference in reverse transcriptase efficiency relative to β-actin.

### 2.6. EROD and GST Activities

The total protein content was determined with the method of Bradford protein assay [27]. EROD activity was determined with the EROD Synthesis Kit (Jianglai, China) according to the manufacturer’s protocol. The samples of the brain and liver were homogenized in an ice-cold homogenization buffer (0.125 M Na_2_HPO_4_∙12H_2_O, 0.125 M KH_2_PO_4_, 0.05 mM Na_2_EDTA, pH = 7.7) and the supernatant was prepared by centrifugation (10,000 rpm, 10 min). The sample mixture (50 μL) was firstly placed in an enzyme-labeled plate and then 100 μL of HRP-labeled enzyme-labeled antibody was added for the reaction at 37 °C for 10 min. After the supernatant was poured, the PBS solution was added and products were washed five times. Reactions were stopped by adding 50 μL of 2 M H_2_SO_4_ into each tube and the supernatant was determined at OD_450._ The GST activities were determined with GST Synthesis Kit (Jianglaibio, China) according to the manufacturer’s protocol. Other steps were similar to those of the EROD kit.

### 2.7. Statistical Analysis

In the statistical analysis, the data were expressed as mean value ± standard deviation (SD). In SPSS 20.0 software (IBM software, Inc., Chicago, IL, USA), one-way ANOVA was carried out. Duncan’s method was used to make the multiple comparisons of the means and *p* < 0.05 indicates a significant difference. All figures were drawn by Excel. A correlation analysis was performed to evaluate the relationship between EROD activity and *CYP1A* mRNA expression, as well as the correlation between GST activity and *GST* mRNA expression.

The Gel imaging analysis system (GIS) can quantify the optical density of DNA bands into a spectrum, and automatically integrate it to calculate the response value. The size of the response value can indirectly reflect the content of DNA. Therefore, the amount of mRNA expression from DNA can be obtained by the inverse calculation.

## 3. Results and Discussion

### 3.1. Median Lethal Concentration (LC50) and Safe Concentration of Carp

According to the experimental procedure of Section 2.3, the acute toxicity dose gradient pre-experiment was first carried out, and the lowest and highest doses of carp death at 24 h and 96 h were recorded: the highest dose of PHE that did not cause death in 96 h was 5 mg/L, the lowest dose of all death in 24 h was 40 mg/L, and when the dose was 20 mg/L, the 96-h mortality rate of carp was about 90%. At 30 mg/L, all carps died within 96 h (Data not shown). Therefore, the acute toxicity dose gradient exposure is set in the range of 5–25 mg/L.

The lethal concentration of PHE for carp are shown in Table 2. With the increase in the concentration of PHE, the mortality of carp also increased, displaying a significant dose-toxicity effect. Furthermore, the regression equations between the probability unit of carp mortality and the logarithmic concentration of PHE after 24-h (Equation (3)) and 96-h (Equation (4)) cultivation were respectively obtained as follows:

Y = 3.970 2X + 0.2275, r^2^ = 0.9388 (3)

Y = 0.479 8X + 0.30, r^2^ = 0.9229 (4)

(Y: Probability unit of mortality; X: logarithmic concentration).

The carp’s LC50 value of PHE after 24 h and 96 h were respectively determined as 15.926 mg/L (95% confidence interval: 14.675–17.282 mg/L) and 11.198 mg/L (95% confidence interval: 9.950–12.604 mg/L). According to the classification standard for acute toxicity of fish [8], PHE is highly toxic to carp. According to Equation (1), the safe concentration of PHE to carp was calculated to be 1.12 mg/L.

### 3.2. Gene Extraction Efficiency

DNA amplification samples were subjected to preset denaturation temperature, cycle parameters, and final repair extension on a gradient PCR instrument. The agarose gel electrophoresis result of 5 μL of samples is shown in Figure S4. The three genes were loaded and electrophoresed simultaneously. From left to right, they were respectively *CYP1A*, *GST*, and *β-ACTIN*, a commonly used internal reference gene. According to the mobility of each DNA and the indication of the marker, the length of DNA fragments in the PCR sample was between 500 bp and 550 bp. Compared with the β-*ACTIN* gene, the *GST* gene fragment was shorter and had a higher electrophoretic mobility, followed by *CYP1A*. The target genes were specifically amplified and showed good integrity. Therefore, the samples could be used for the semi-quantitative analysis, recovery, and sequencing for further gene comparison.

The sequencing and splicing results of *CYP1A* and *GST* genes and alignment results obtained from the gene bank (NCBI, National Center for Biotechnology Information) are shown in Table 3. The lengths of the two amplified genes (*CYP1A* and *GST*) were respectively 503 bp and 541 bp. Similarly, the amplified gene had a high similarity of greater than 99% to the target gene in the gene bank. Therefore, the entire RNA extraction, reverse transcription and PCR operations could be qualified.

### 3.3. CYP1A mRNA Expression and EROD Activity after the Exposure to PHE

**Figure 1:**
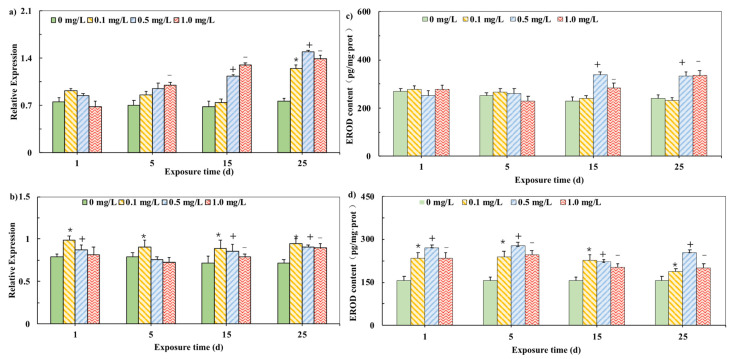
Relative mRNA expression of *CYP1A* in liver (**a**) and brain (**b**) of carp exposed to PHE. Effects of different PHE concentrations on EROD activities in carp (**c**) in the liver. (**d**) in the brain (The final expression results are based on the content of characteristic proteins in the mass of each tissue unit of the carp.). Expression was quantified by Sq-RT-PCR. Values are mean ± SD of three replicates. Significant differences, *p* < 0.05. (*) are under 0.1 mg/L versus negative control of 1 d; (+) are under 0.5 mg/L versus negative control of 1 d; (−) are under 1.0 mg/L versus negative control of 1 d, which is based on Duncan’s method.

The *CYP1A* mRNA expression in carp tissues are showed in Figure 1. We examined the effect of 0 mg/L PHE in the brain and liver as negative control. Pearson correlation indicated the significant correlation between *CYP1A* mRNA concentration and time in both brain and liver. *CYP1A* mRNA expression levels in the liver showed a significant increase compared with those in the control group during 25-day exposure to PHE (Figure 1a) and the induction trend gradually became more significant during the exposure. *CYP1A* mRNA levels were induced significantly by 0.50 mg/L PHE during the 25-day exposure. In the brain, *CYP1A* mRNA levels were significant induced compared with those in the control, but the induction effect was not significantly enhanced with the increase in exposure time or doping concentration (Figure 1b).

To detect whether the change in *CYP1A* mRNA brought about the change of related metabolic characteristic enzymes, the enzyme activity of EROD was tested (Figure 1) We examined the effect of 0 mg/L PHE in the brain and liver as negative control. In the liver, the EROD activity did not change significantly in the initial stage, but all experimental groups resulted in a significant EROD induction in the liver in the 15th day and 25th day (Figure 1c). In the brain, the activity of EROD in the early period of PHE exposure (Day 1, 5 and 15) was significantly induced compared with the control assays, (*p* < 0.05). However, on Day 25, the induction was not significant (Figure 1d). The activity induction trend of the EROD enzyme in the liver and brain is consistent with the mRNA expression trend of *CYP1A*. It shows that long-term exposure of carp to a safe concentration of PHE will stimulate the expression of *CYP1A* mRNA in the liver of carp and the increase in mRNA expression of *CYP1A* reflects an increase in protein functionality [28].

It widely reported that when an organism is exposed to pollutants such as heavy metals and persistent organic pollutants (POPs), the activity of antioxidant enzymes will be affected [29]. Cytochrome P450 enzyme (CYP450) is widely present in organisms and is an important metabolic enzyme involved in the conversion of many heterologous compounds [30]. CYP1A is a member of the cytochrome P450 family. CYP1A enzyme activity is considered to be an indicator of fish resistance to PAH.

Previous studies have shown that mild oxidative stress can induce the activity of antioxidant enzymes such as SOD and GST [31]. However, when oxidative stress reaches a certain level, the activity of antioxidant enzymes will be inhibited [32]. In this study, the expression of *CYP1A* mRNA and the activity of EROD enzyme in the liver and brain increased with time and concentration. Moreover, the level of induction in the liver is more pronounced than in the brain. The liver is a key site of detoxification and an important target organ of PAHs [33]. EROD is the important enzyme assisting in the metabolism of toxic compounds. If pollutants enter the body, the organism starts stress reactions. The differential responses of transcription and expression of characteristic genes can be used as early warning parameters to measure the degree of environmental pollution. The change in *CYP1A* mRNA expression in the liver and brain of carp was the consequence of the stimulation of the signaling pathway. If the concentration of PHE was too high, it might cause damage and inhibit the gene expression and enzyme activity [20]. Similar to previous reports, after intraperitoneal injection of TCDD in goldfish, it was also found that the expression of *CYP1A* mRNA was induced in various tissues, and the induction effect was most significant in liver tissues. It was reported that after the exposure of medaka to pentachlorobiphenyl, CYP1B1 and CYP1C1 mRNA expressions were induced due to the acceleration of Phase I metabolic response [34]. If a pollutant entered the carp body, the organism could defend itself by stimulating the production of Phase I- and Phase II-related enzymes degrading external compounds. However, if excessive external substances existed, the body defense mechanism became destroyed.

A similar phenomenon was observed in the liver of tilapia (*Oreochromis niloticus*). The short-term exposure to a low concentration of PHE induced EROD activities in the liver of tilapia *(Oreochromis niloticus)*, whereas the long-term exposure to a high concentration of PHE inhibited EROD activities [35]. It was also found that several PAHs with lower molecular weights caused the differential expressions of P450 enzymes [36]. It was also reported that a low concentration of PHE activated EROD in young *Sparus aurata*, but inhibited it under high concentration [37]. In the brain and liver of carp, the activity of EROD showed an obvious correlation with the expression level of *CYP1A*. Many compounds could induce EROD activity of fishes and the induction of EROD activity might be impeded as chemicals were competitively bound to the structure of AhR or *CYP1A* [38].

### 3.4. GST mRNA Expression and GST Activity after the Exposure to PHE

**Figure 2:**
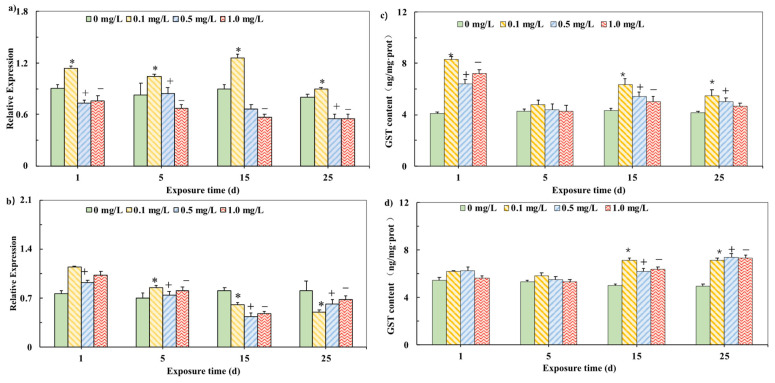
Relative mRNA expression of *GST* in liver (**a**) and brain (**b**) of carp exposed to PHE. Effects of different PHE concentrations on GST activities in the liver (**c**). and the brains (**d**) of carp (The final expression results are based on the content of characteristic proteins in the mass of each tissue unit of the carp.) Expression was quantified by Sq-RT-PCR. Values are mean ± SD of three replicates. Significant differences, *p* < 0.05 [26]. (*) are under 0.1 mg/L versus negative control of 1 d; (+) are under 0.5 mg/L versus negative control of 1 d; (−) are under 1.0 mg/L versus negative control of 1 d, which is based on Duncan’s method.

The *GST* mRNA expressions in carp tissues are shown in Figure 2. In the liver, the expression of *GST* mRNA was always induced when carp was exposed to 0.1 mg/L PHE and the induction effect was weakened in the 25th day. However, the exposure to 0.5 and 1.0 mg/L PHE showed a significant inhibition effect on *GST* mRNA expression from the first day to the 25th day (Figure 2a). In the brain, the experimental groups of different concentrations showed the significant induction in the first day and the induction effect was reduced in the 5th day. During the PHE exposure period, the expression of *GST* mRNA showed a significant inhibitory effect in the 15th day and the 25th day (Figure 2b).

In order to detect whether the change in *GST* mRNA level brought about the change in related characteristic metabolic enzymes, the enzyme activity of GST was tested (Figure 2). In the liver, the GST activities of all experimental groups were significantly stimulated after induction (*p* < 0.05), but the induction effect was gradually weakened (Figure 2c). There was no obvious change in the GST activity at the beginning of the experiment (from the first day to the 5th day) in the brain. However, during the PHE exposure period, the GST activities in the 15th and 25th days had a significant induction effect (*p* < 0.05) (Figure 2d).

GST can catalyze the binding of exogenous compounds such as insecticides, herbicides, and antibiotics to glutathione, and avoids damaging other cell biomolecules, thus playing an important role in combating oxidative stress [39]. In this study, the GST enzyme activity of the liver and brain were both induced by exposure to lower than safe concentrations of phenanthrene. This is similar to the previous report: The reports found that five kinds of PAHs induced the GST activity in the carp liver [40]. Yin et al. also reported that the GST activity in the tissues of catfish was significantly increased after the exposure to PHE [41]. Nahrgang et al. reported that benzo (a) pyrene induced the GST activity in cod liver [20]. However, Olinga et al. found that the short-term exposure to PHE inhibited GST activity in the kidney of *Liza aurata* [42].

However, the expression of mRNA in the liver and brain of carp is not synchronized with the changes in GST enzyme activity. mRNA expression in the liver was inhibited at concentrations of 0.5 and 1.0 mg/L. mRNA expression in the brain is first induced and then suppressed over time. This phenomenon of asynchrony between enzyme activity and gene expression also exists in previous reports: Sun et al. reported that after yellow catfish was exposed to Hg^+^, the related *SOD* and *GPX1* mRNA expression levels increased significantly, but the SOD enzyme and GPX1 enzyme activities did not change much [43]. The analysis of the cause of this phenomenon may be due to the fact that antioxidant enzymes such as GST are encoded by multiple subtype genes. However, semi-quantitative genetic testing only analyzes one subtype gene, so changes in mRNA levels do not necessarily occur at the enzyme level. On the other hand, there is a time lag effect in the process of gene transcription, translation and protein modification, so the enzyme activity cannot reflect the mRNA level in time [44]. In the study, the expression of *GST* mRNA in carp liver and brain showed the early induction and subsequent inhibition effects, indicating that after the Phase I metabolic reactions, the biological activity of GST in the tissues of carp gradually became unbalanced during the exposure.

### 3.5. Correlation Analysis

**Figure 3:**
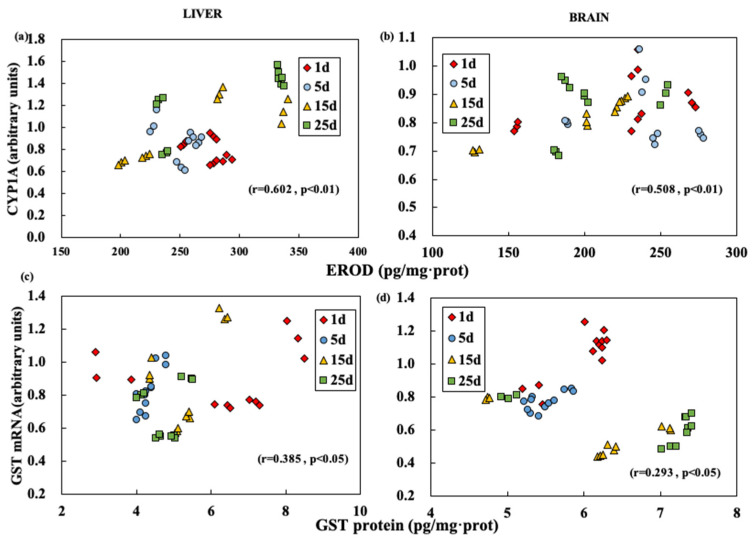
Correlation of EROD and *CYP1A* mRNA (**a**,**b**), GST and *GST* mRNA (**c**,**d**) values in brain (**b**,**d**) and liver (**a**,**c**) at different exposure time.

The correlation analysis was carried out to test the expression levels of characteristic enzymes and characteristic mRNA (Figure 3). The correlation coefficient indicates that EROD activity in the liver showed a positive correlation with *CYP1A* mRNA level (r = 0.602, *p* < 0.01) (Figure 3a). In the brain, the correlation between EROD activity and *CYP1A* mRNA level was less significant than that in the liver (r = 0.508, *p* < 0.01) (Figure 3b). However, in the liver or brain, the correlation between GST activity and *GST* mRNA expression was relatively poor (liver, r = 0.395, *p* < 0.05; brain, r = 0.293, *p* < 0.05) (Figure 3c,d).

In the study, the activity of EROD in the liver was more highly correlated with the activity of *CYP1A* mRNA expression. The liver is an important metabolism organ of heterogeneous organisms and the blood mediates the relationship between the brain and other target organs and accelerates the reaction between the characteristic liver enzymes and its mRNA expression. Similar experimental results had been reported in previous studies [7,45]. After the brain and muscle were exposed to soluble components of crude oil, the changes in AchE activity were not obvious compared to other organs [46]. In addition, the toxicity induced by CYP1A indicated the chemical exposure and preferential effects in various biological tissues [38].

The cytochrome P450 system showed a highly correlation than the GST system in both the liver and brain. The cytochrome P450 enzyme system played an important role in the heterogeneous biotransformation in Phase I metabolism of fish and other aquatic animals. The activity of ethoxysalolin O-deethylase (EROD) and the *CYP1A* mRNA level seemed to be the most sensitive catalytic probe. The induction response of the cytochrome P450 system had been determined in many studies [47,48]. The GST is a representative enzyme in Phase II metabolism and play an important role of catalyzing or reducing oxidative substances in the metabolic body. In most cases, only the modest changes in total GST activity were reported [49]. Similar studies reported that *GSTα* mRNA expression in all tissues showed no significant difference compared with *CYP1A* and ABC efflux transporters after the exposure to benzo(*a*)pyrene [45].

In addition, there was a significant decrease in *GST* mRNA in the brain after 15 days of PHE exposure. The higher GST activity and the lower *GST* mRNA expression in the 15th day might be interpreted as follows. From the first day to the 5th day, the activity of GST increased, thus increasing the clearance rate of PHE and reducing the overall mRNA response and *GST* synthesis. As a result, the mRNA expression level was reduced (Figure 2). However, the same phenomenon was not observed in the liver, indicating the differences in the heterogeneous metabolism and PHE-induced enzymes between the brain and liver [20].

### 3.6. Mechanism

**Figure 4:**
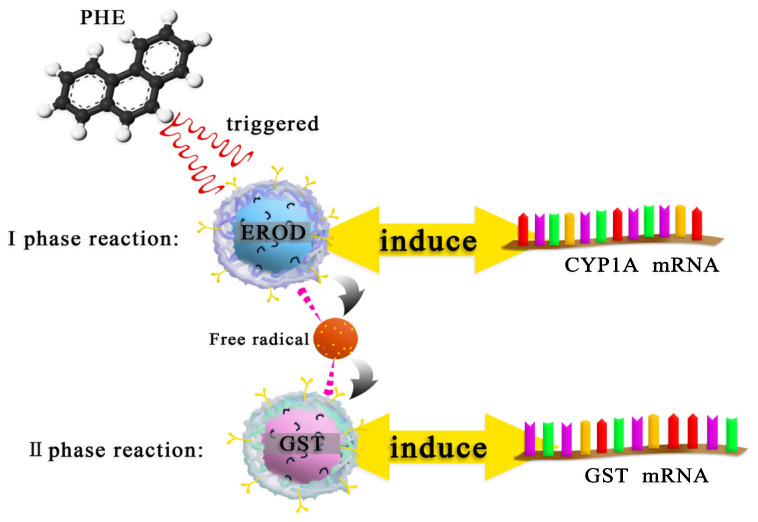
The metabolic mechanism of PHE exposed to carp.

The possible mechanism of PHE exposed to carp is shown in Figure 4. When PHE entered the carp body, the characteristic enzymes of the I-phase reaction were activated to remove pollutants, which resulted in an increase in the expression of *CYP1A* gene. As the body’s defense oxidation system proceeds to the II-phase reaction, the expression of GST and *GST* genes is stimulated.

## 4. Conclusions

In conclusion, our study used carp as the test organism to perform acute toxicity tests and calculate safe concentrations. Exposure of carp to sub-safe concentrations of PHE for 25 days resulted in changes in oxidative stress. The expression of EROD and GST enzymes in the liver was stimulated from day 1 of exposure to induce the expression of *CYP1A* and *GST* genes. The expression activities of EROD and GST enzymes in the brain were stimulated, but the expression of *GST* gene was delayed. Correlation analysis also illustrates this phenomenon. These results suggest that long-term exposure of carp to sub-safe concentrations still affects its oxidase system and gene expression. The regulation of carp’s own molecular level under the stress of exogenous pollutants was revealed.

## Figures and Tables

**Table 1:** Semi-quantitative RT-PCR (Sq-RT-PCR)primers of *Cyprinus carpio* genes.

Gene	GeneBank Number	Primer Sequence	Product Size [11]
β-actin	AF0570 40	F: CCATCTACGAGGGTTACGCC	551 bp
		R: AATGCCAGGGTACATGGTGG	
*CYP1A*	AB048939.1	F: CTGAGCCTGACCGCTATGAG	503 bp
		R: CCGCTTCCTACGATCTTCCC	
*GSTs*	LC071505.1	F: CCGCTTCCTACGATCTTCCC	541 bp

**Table 2:** LC50 of *Cyprinus carpio* after 24-h and 96-h exposure to PHE.

Variable	Lethal Concentration							
Concentration/(mg·L^−1^)	5.01	6.31	7.94	10.00	12.59	15.85	19.95	25.12
Log concentration	0.7	0.8	0.9	1.0	1.1	1.2	1.3	1.4
24 h mortality rate/%	0	0	20	20	30	60	70	70
Unit of probability	3.04	3.04	4.16	4.16	4.48	5.25	5.52	5.52
96 h mortality rate/%	10	20	20	30	40	70	90	100
Unit of probability	3.72	4.16	4.16	4.48	4.75	5.52	6.28	6.96

**Table 3:** Alignment results of sequenced results of amplified target genes ^a^.

Genes	Gene Length/bp	Accession No. ^b^	Gene Similarity/%
*CYP1A*	503	AB048939.1	99.7
*GST*	541	LC071505.1	99.5

^a^ The splicing results and peak images of the sequencing results are shown in the supplementary information. ^b^ The serial number is the gene number in GeneBank (NCBI).

## Data Availability

Data available on request due to restrictions eg privacy or ethical The data presented in this study are available on request from the corresponding author. The data are not publicly available due to confidentiality agreement.

## Supplementary Materials

The following supporting information can be downloaded at: https://www.mdpi.com/article/10.3390/ijerph19042129/s1, Figure S1: Electrophoresis diagram of RNA sample (Lanes 1 and 2 indicate the electrophoresis of liver and brain RNA of carp domesticated in clean water, and lanes 3 and 4 are the extraction of total RNA from tissues after PHE stress. Figure S2: GST amplified gene sequencing stitching results. Figure S3: CYP1A amplified gene sequencing stitching results. Figure S4: Electrophoresis diagram of each gene sample. The expression levels of target genes CYP1A and GST were detected by genomic PCR analysis, and β-ACTIN was used as a template control. From top to bottom are the cDNA gel electrophoresis diagrams of PHE stress concentrations of 0, 0.1, 0.5, 1.0 mg/L.

## Abbreviations

AhR	aromatic hydrocarbon receptors
BSA	Albumin from bovine serum
CYP1A	cytochrome P4501A
DEPC	Diethy pyrocarbonate
EROD	7-Ethoxylesorufin O-deethylase
GST	glutathione S-transferase
PAHs	polycyclic aromatic hydrocarbons
PBS	Phosphate buffer solution
PHE	Phenanthrene
POPs	persistent organic pollutants
Sq-RT-PCR	semi-quantitative reverse transcription and polymerase Chain reaction
