# Supplementary material for: Long-Term Exposure to Phenanthrene Induced Gene Expressions and Enzyme Activities of Cyprinus carpio below the Safe Concentration
Source: Int J Environ Res Public Health. 2022 Feb 14;19(4):2129. doi: 10.3390/ijerph19042129 (PMC8872569; doi:10.3390/ijerph19042129)

Supporting Information for

Long-Term Exposure to Phenanthrene Induced Gene Expressions and Enzyme Activities of *Cyprinus Carpio* below the Safe Concentration

Xin Kang [1], Dongpeng Li [1], Xiaoxiang Zhao [1], Yanfeng Lv [1], Xi Chen [2], Xinshan Song [1], Xiangyu Liu [3], Chengrong Chen [3] and Xin Cao [1,*]

[1] Textile Pollution Controlling Engineering Center of Ministry of Environmental Protection, College of Environmental Science and Engineering, Donghua University, Shanghai 201620, China; cathy3449@163.com (X.K.); lddest@163.com (D.L.); zxx@dhu.edu.cn (X.Z.); lvyf_up@outlook.com (Y.L.); newmountain@163.com (X.S.)

[2] Agricultural Genomics Institute at Shenzhen, Chinese Academy of Agricultural Sciences, Shenzhen 518124, China; chenxi02@caas.cn

[3] Australian Rivers Institute and School of Environment and Science, Griffith University, Nathan, QLD 4111, Australia; xiangyu.liu2@griffithuni.edu.au (X.L.); c.chen@griffith.edu.au (C.C.)

[*] Correspondence: caoxin@dhu.edu.cn; Tel.: +86-21-6779-2550

Supporting Information: 4 Figures.

Figure S1. Electrophoresis diagram of RNA sample (Lanes 1 and 2 indicate the electrophoresis of liver and brain RNA of carp domesticated in clean water, and lanes 3 and 4 are the extraction of total RNA from tissues after PHE stress. The time shown in Fig. is the time when the three characteristic bands of the sample RNA electrophoresis appear.)

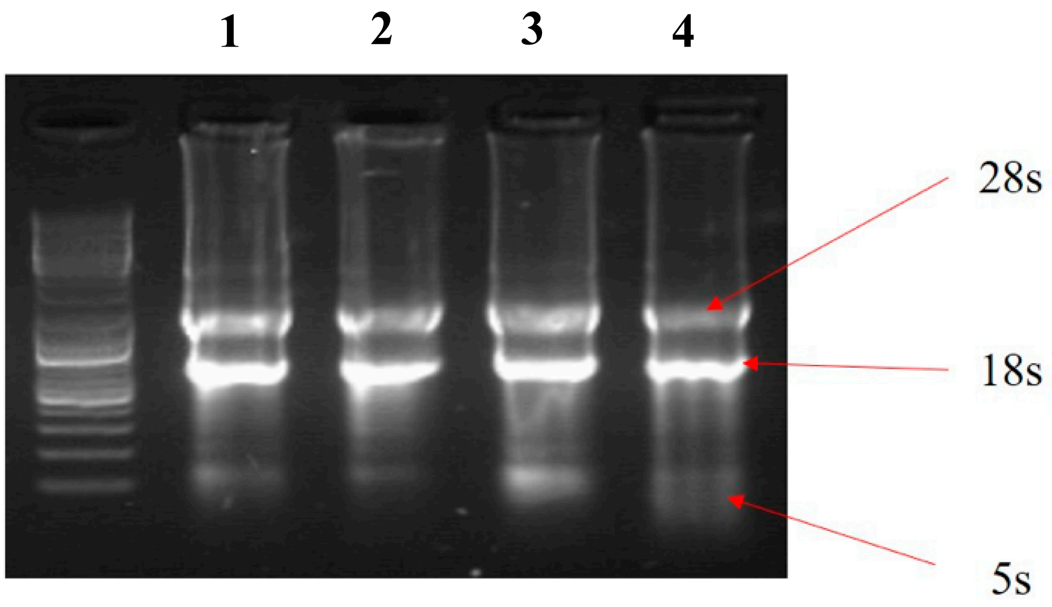

Figure S2. GST amplified gene sequencing stitching results

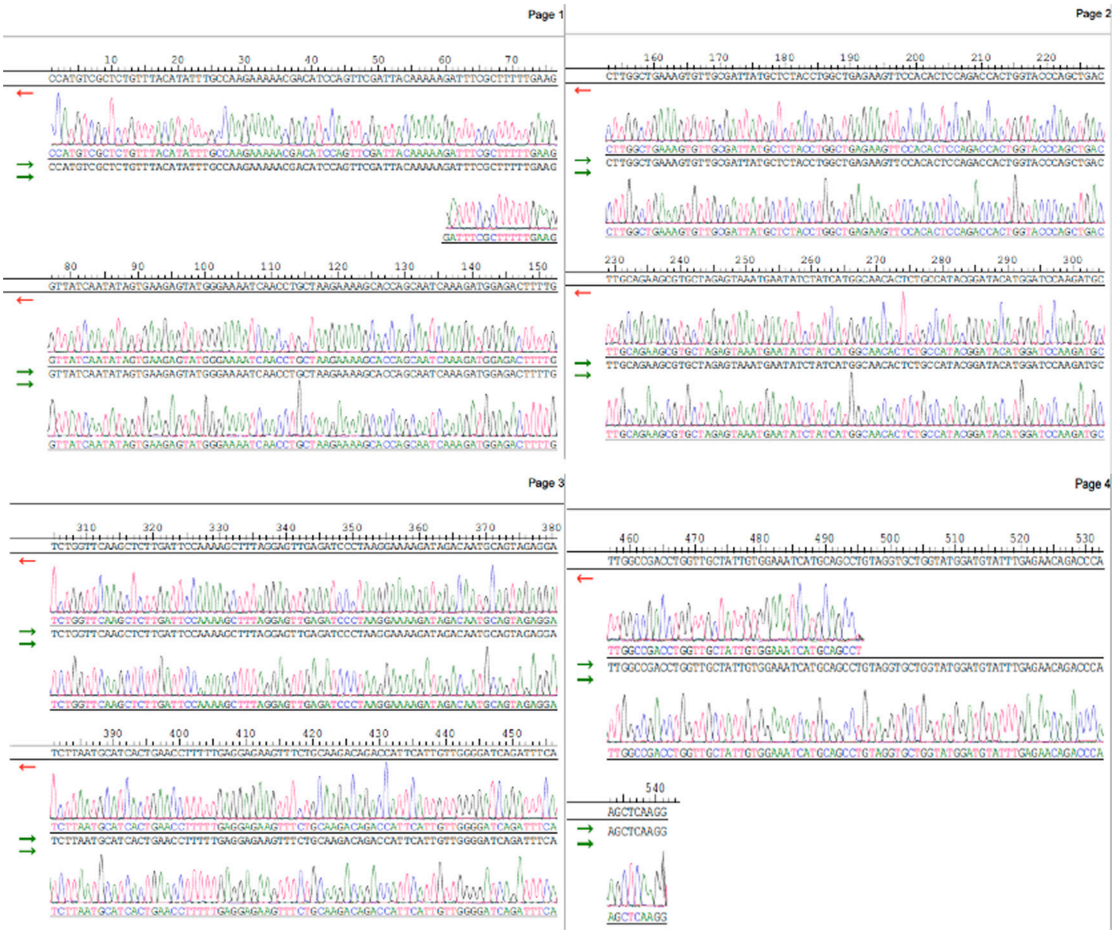

Figure S3. CYP1A amplified gene sequencing stitching results

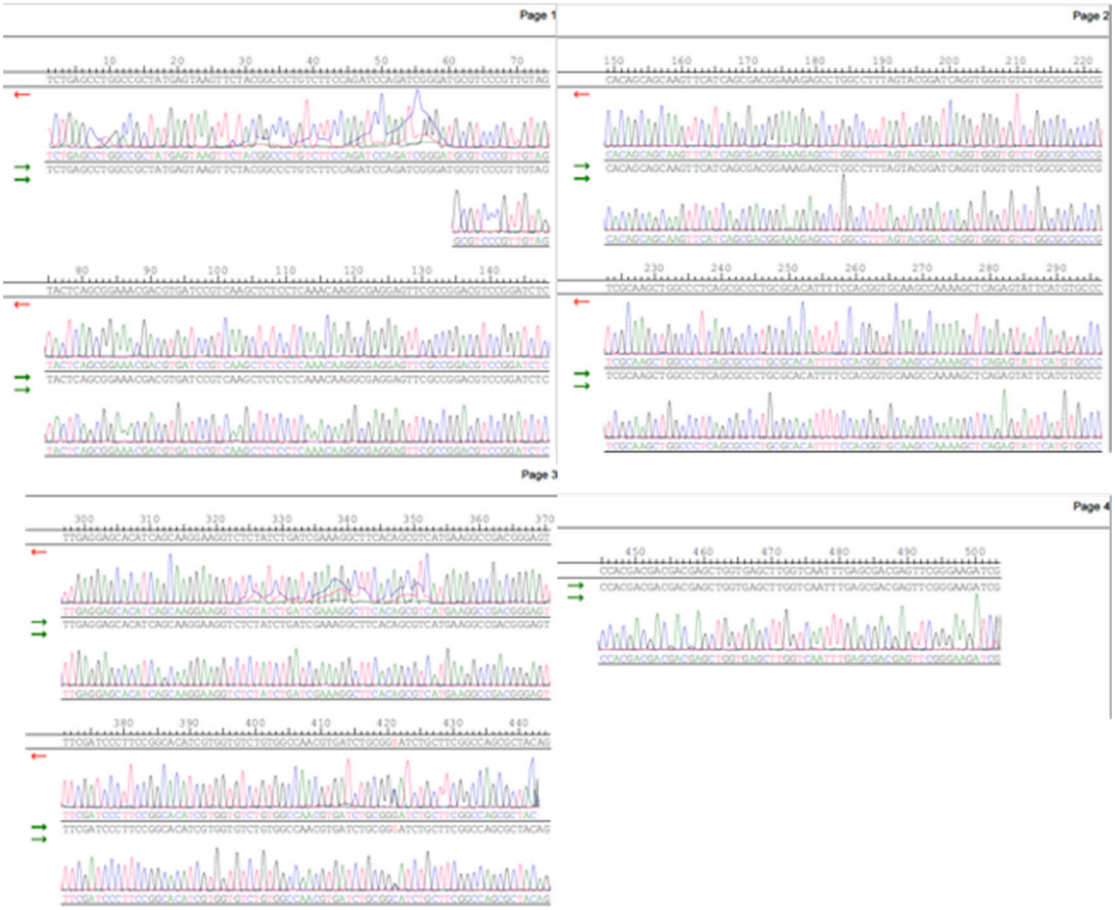

Figure S4. Electrophoresis diagram of each gene sample. The expression levels of target genes CYP1A and GST were detected by genomic PCR analysis, and β-ACTIN was used as a template control. From top to bottom are the cDNA gel electrophoresis diagrams of PHE stress concentrations of 0, 0.1, 0.5, 1.0 mg/L.

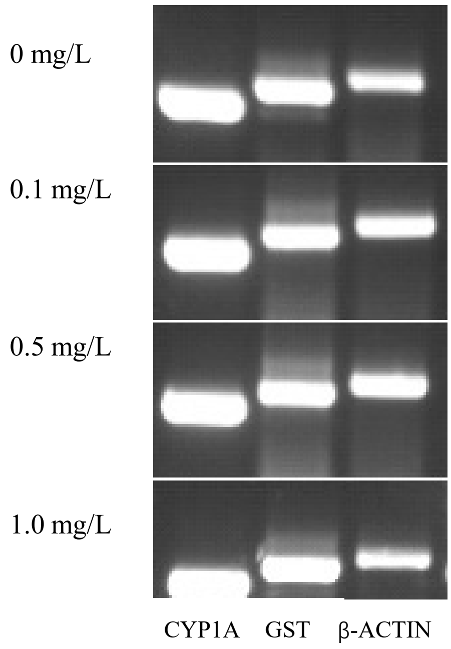

Supplement: Supplementary file 1 [file ijerph-19-02129-s001.zip › ijerph-1521540-supplementary.pdf]
